# Blocking H2A.Z Incorporation via Tip60 Inhibition Promotes Systems Consolidation of Fear Memory in Mice

**DOI:** 10.1523/ENEURO.0378-18.2018

**Published:** 2018-11-08

**Authors:** Klotilda Narkaj, Gilda Stefanelli, Malak Wahdan, Amber B. Azam, Firyal Ramzan, Carl Frank David Steininger, Brandon J. Walters, Iva B. Zovkic

**Affiliations:** 1 Department of Cell and Systems Biology, University of Toronto, Toronto, Ontario M5S 3G5, Canada; 2 Department of Psychology, University of Toronto Mississauga, Mississauga, Ontario L5L 1C6, Canada; 3 Department of Neurosciences & Mental Health, the Hospital for Sick Children, Toronto, Ontario M5G 1X8, Canada

**Keywords:** chromatin, epigenetics, histone variants, H2A.Z, Tip60, histone acetylation

## Abstract

Memory formation is a protracted process that initially involves the hippocampus and becomes increasingly dependent on the cortex over time, but the mechanisms of this transfer are unclear. We recently showed that hippocampal depletion of the histone variant H2A.Z enhances both recent and remote memories, but the use of virally mediated depletion reduced H2A.Z levels throughout testing, making its temporally specific function unclear. Given the lack of drugs that target histone variants, we tested existing drugs for efficacy against H2A.Z based on their targeting of known H2A.Z regulators. The Tip60 (part of H2A.Z deposition complex) inhibitor Nu9056 reduced H2A.Z binding, whereas the histone deacetylase (HDAC) inhibitor Trichostatin-A increased H2A.Z acetylation without influencing total H2A.Z in cultured hippocampal neurons. Tip60 (but not HDAC) inhibition 23 h after learning enhanced remote (tested at 7 d) and not recent (tested at 24 h) contextual fear memory in mice. In contrast, Tip60 inhibition 30 d after learning impaired recall of remote memory after 1 h, but protected the memory from further decline 24 h later. These data provide the first evidence of a delayed postlearning role for histone variants in supporting memory transfer during systems consolidation.

## Significance Statement

Understanding the molecular basis of memory is fundamental for developing novel therapies for memory decline, but progress is impeded by the presence of multiple stages of memory formation that may involve unique molecular events. Our data identify a new time point, 24 h after learning, at which time H2A.Z manipulation selectively impacts remote memory without influencing recent memory. This finding advances our understanding of memory for several reasons, as follows: (1) it demonstrates that ongoing epigenetic changes exert stage-specific outcomes on memory formation; and (2) it provides the first pharmacological tool for manipulating H2A.Z binding. Moreover, we show that Tip60 influences memory through effects that involve H2A.Z, which suggests that recent studies of Tip60-mediated plasticity may include actions on this histone.

## Introduction

Memory deficits are common across various forms of dementia and neuropsychiatric conditions, but effective treatments are lacking. One contributing factor is the complexity of memory formation, both in terms of molecular mechanisms and the time scales at which memories form. Memory formation is a multistage process that begins with transient synaptic consolidation in the hippocampus, which lasts ∼3 h and is the focus of most studies (Walters and Zovkic, 2015). This stage is accompanied by a prolonged period of systems consolidation (at least 7 d), during which memories are gradually transferred from short-term storage in the hippocampus for long-term maintenance in the cortex (Frankland and Bontempi, 2005; Lesburguères et al., 2011).

In contrast to the growing understanding of the initial consolidation phase within the first 2 h after learning, the molecular steps that underlie the later stages of memory formation and maintenance are poorly understood. Several studies indicate that delayed waves of hippocampal gene expression ≥8 h after learning, selectively impact remote over recent memory (Taubenfeld et al., 2001; Merhav et al., 2006; Bekinschtein et al., 2007; Katche et al., 2010, 2012), indicating that delayed time points may be critical for creating long-lasting memories. As such, a better understanding of this process is essential for developing novel interventions for memory decline.

Epigenetic mechanisms are implicated in all stages of memory, with DNA methylation emerging as the primary candidate for memory maintenance in the cortex (Miller et al., 2010; Halder et al., 2016). In contrast, changes in post-translational histone modifications occur rapidly after learning and return to baseline shortly thereafter (Peixoto and Abel, 2013; Halder et al., 2016), but may nevertheless be involved in stabilizing remote memory (Lesburguères et al., 2011). Several recent studies showed that DNA and histone methylation in the hippocampus are modified 24 h after learning (Rasmussen et al., 1999; Gupta et al., 2010; Gupta-Agarwal et al., 2012), indicating a potential role for epigenetics in ongoing systems consolidation, but the functional implications of these delayed changes in memory have not been investigated.

We recently found that histone variants, which replace canonical histones in chromatin, are novel epigenetic regulators of memory (Zovkic et al., 2014), whereby the histone variant H2A.Z is a memory suppressor that is rapidly evicted from hippocampal and cortical chromatin after learning (Zovkic et al., 2014; Stefanelli et al., 2018). Whereas hippocampal H2A.Z depletion improved both recent and remote memory, cortical H2A.Z depletion improved only remote memory, suggesting that hippocampal H2A.Z may regulate both stages of memory. Moreover, cortical H2A.Z binding is altered 7 d after learning (Zovkic et al., 2014), suggesting that H2A.Z has the capacity to persist over time. Based on this observation and the evidence that delayed gene expression selectively regulates memory recall of older memories, recalled 7 d after learning (Katche et al., 2010, 2012, 2013), we hypothesized that hippocampal H2A.Z binding is altered after 24 h to selectively support systems consolidation. Given that H2A.Z is subject to acetylation that modifies its effects on chromatin (Ku et al., 2012; Valdés-Mora et al., 2012), we also investigated changes in acetylated H2A.Z.

To determine the functional relevance of H2A.Z and its acetylation at 24 h, we sought a novel method to rapidly manipulate H2A.Z binding, as our typical approach includes viral H2A.Z depletion across all stages of memory formation and recall (Zovkic et al., 2014; Stefanelli et al., 2018). To this end, we showed that Tip60 (part of complex involved in H2A.Z deposition) inhibition via Nu9056 effectively reduced H2A.Z levels, whereas histone deacetylase (HDAC) inhibition via Trichostatin-A (TSA) promoted H2A.Z acetylation without influencing total H2A.Z. These drugs allowed us to achieve temporally specific inhibition of H2A.Z or acetylated H2A.Z (acH2A.Z) to tease apart their effects on recent (24 h) versus older (7 d) memory and remote (30 d) memory. The 7 d time point was selected because it represents an intermediate time point between recent and remote memory, when memories become increasingly independent of the hippocampus (Frankland and Bontempi, 2005; Zovkic et al., 2014; Walters and Zovkic, 2015). Given that H2A.Z is a memory suppressor whose levels accumulate in the aging hippocampus (Stefanelli et al., 2018), the identification of drugs that effectively manipulate H2A.Z levels have therapeutic implications for memory decline.

## Materials and Methods

### Animals

Male C57BL/6J mice were bred in our colony at the University of Toronto Mississauga (UTM) and housed in groups after weaning at 21 d, with ad libitum access to food and water and a 12 h light cycle (lights on at 8:00 A.M.). Testing began when mice reached adulthood, ranging between 9 and 12 weeks of age. All procedures were approved by the UTM Animal Care Committee and complied with institutional guidelines and the Canadian Council on Animal Care.

### Fear conditioning

Mice were habituated to handling and testing for 3 d before fear conditioning, whereby mice were transferred from the colony to the testing room for 30 s of handling daily. To measured learning-induced changes in H2A.Z and acH2A.Z binding, the mice were assigned to one of the following four groups: Naive (N), Context (C), Shock (S), or Context and Shock (CS). All mice, except for those in the naive group, were placed in the training chamber and given 2 min to explore, followed by five electric footshocks (0.7 mA, 2 s) administered 1 min apart to the CS group, with an additional minute of exploration before removal from the chamber. Mice in the C group underwent the same procedure without the footshock, whereas mice in the S group were rapidly shocked in the dark to avoid context learning. N mice remained in their home cage until tissue collection 24 h or 30 d after training.

Mice used for behavioral studies were assigned to one of the following two groups: vehicle or Nu9056/TSA, with the assigned drug depending on the experiment. Drug injections occurred 1 h before testing, based on wide usage of this time point for administering HDAC inhibitors in fear conditioning experiments. All mice were placed in the training chamber and given 2 min to explore before receiving a single 0.5 mA (2 s) footshock and an additional minute of exploration. This weaker training protocol was used here to prevent ceiling effects in our behavioral studies. Twenty-three hours after training, mice were injected with the assigned drug and placed back into the training chamber for a 3 min recall test 24 h (i.e., 1 h after injection) and/or 7 d after training, depending on the experiment. Memory was quantified as the percentage of time spent freezing, determined through automatic scoring using FreezeFrame Software (ActiMetrics).

### Drug preparation

The Tip60 inhibitor, Nu9056 (10 μg/μl in DMSO; Tocris Bioscience) was diluted to a working solution of 0.5 μg/μl in saline, and an injection dose of 2 μg/g was achieved by injecting 4 μl/g. The HDAC inhibitor TSA (1.21 μg/μl in DMSO; Cell Signaling Technology) was diluted to a working solution of 0.075 μg/μl in saline, and an injection dose of 0.3 μg/g mouse was achieved by injecting 4 μl/g.

### Primary hippocampal neurons and drug treatments

Hippocampi were isolated from embryonic day 17 pups, washed in HBSS, digested with trypsin 0.25% (Thermo Fisher Scientific), and mechanically dissociated by pipetting. Cells were plated on poly-l-lysine (0.1 mg/ml; Sigma-Aldrich) at a density of 250,000/well in a six-multiwell plate. Neurons were grown in a culture medium containing Neurobasal (Thermo Fisher Scientific), B_27_ (Thermo Fisher Scientific), and l-glutamine (Thermo Fisher Scientific). After 14 d, cells were treated as follows: for effects of Tip60 inhibition, Nu9056 was added at a final concentration of 400 nm for 2 h, with the appropriate amount of DMSO used as control. For effects of HDAC inhibition, TSA was added to a final concentration of 250 nm for 4 h, with the appropriate amount of DMSO used as a control.

### Protein extraction and Western blotting

Hippocampal tissues were homogenized using a Dounce homogenizer in RIPA buffer (50 mm Tris HCl, pH 7.4; 150 mm NaCl; 10% NP-40; 0.5% sodium deoxycholate; 0.1% SDS) supplemented with Protease Inhibitor Cocktail (Cell Signaling Technology). Homogenates were incubated for 20 min on ice, centrifuged at maximum speed (13,000 rpm) at 4°C for 15 min, and the supernatant was collected and quantified using the Bradford assay (BioShop). For primary hippocampal neurons, the medium was removed and wells were washed with HBSS (Thermo Fisher Scientific). One hundred microliters of 6× sample buffer was added to each well, cells were scraped, and lysates were collected. Equal volumes of each sample were loaded on the gel.

Proteins were separated on 15% SDS-PAGE and transferred to PVDF membrane. Membranes were blocked in TBST (10 mm Tris–HCl, pH 8.0; 150 mM NaCl; and 0.05% Tween 20) containing 5% milk and incubated with primary antibodies overnight at 4°C. The following primary antibodies were used: H2A.Z (1:1000; catalog #ABE1348, Millipore), acH2A.Z (1:1000; catalog #ABE1363, Millipore), and β-actin (1:2000; catalog #4967S, Cell Signaling Technology). Membranes were incubated with appropriate secondary antibody [1:10,000; catalog #707P2 (mouse), catalog #7074S (rabbit), Cell Signaling Technology] for 1 h at room temperature, and detection was performed by enhanced chemiluminescence (Thermo Fisher Scientific). Images were analyzed with ImageJ (National Institutes of Health) and normalized to the appropriate actin control. For the immunoblots seen in Figure 2, the membrane was stripped (0.1 m Tris-HCl, pH 6.8; 2% SDS; 0.7% β-mercaptoethanol) after staining for H2A.Z and reprobed for acH2A.Z. For the remaining blots, H2A.Z and acH2A.Z were run on separate gels and normalized to corresponding actin on each membrane.

### Chromatin immunoprecipitation

Briefly, hippocampi or primary hippocampal neurons were incubated in 1% formaldehyde for 10 min at 37°C, when 1.25 m glycine was added to quench the reaction. Next, samples were washed with PBS, and SDS lysis buffer was added to all samples before sonication (40% power, 6× for 10 s with 50 s of rest; Thermo Fisher Scientific). Samples were centrifuged at 17,000 × *g* for 10 min, aliquoted, and diluted with chromatin immunoprecipitation (ChIP) dilution buffer (Millipore). Samples were treated with 20 μl of Millipore Protein G magnetic beads and 1 μl of H2A.Z (catalog #ABE1348, Millipore), AcH2A.Z (catalog #ABE1363, Millipore), or H3 (catalog #05-499, Millipore) antibody overnight at 4°C. The next day, samples were washed sequentially with low-salt, high-salt, LiCl (Millipore), and Tris-EDTA (TE) buffer and incubated with rotation for 5 min between washes. Immune complexes were extracted using TE buffer and proteinase K (for both ChIP and input samples) and heated at 65°C for 2 h, followed by 95°C for 10 min before purification with a PCR Purification Kit (Bio Basic). Primers were designed to detect specific sequences (Table 1), and ChIP data were calculated as the percentage of input, then normalized against the control group.

**Table 1:** List of primers used for ChIP PCR

	**Forward sequence**	**Reverse sequence**
+1 Arc	5′-GCCACACTCGCTAAGCTCC	5′-AACTCCTCTGAGGCAGAAGCC
−1 Arc	5′-TCCCGGTGGGAGGCG	5′-GTGCCCTCAAGGACCCG
+1 Fos −1 Fos +1 Gria4 -1 Gria4	5′-AGTGTCTACCCCTGGACCC 5′-AGGAGACCCCCTAAGATCCC 5′-CCATGGGGAGGTGCCTAACTT 5′-TTCTAGACCCCAGCCTCATCA	5′-GCGTTGAAACCCGAGAACATC 5′-CTGTCGTCAACTCTACGCCC 5′-GCTTGGCAGGAAACTACCCC 5′-CGGATGGATGCGGTAAAGGTA
+1 Kcna2 -1 Kcna2	5′-GGATTAGACATCCAGCTCAGG 5-GATGTGCAAGCGAAGAACCC	5′-CTCCCCCATCTCTCTTGGTA 5′-GAGATGGCAGCACTGGCT
+1 Syt1 -1 Syt1	5′-GGGATGCTCTGACCGAGTTC 5′-TGGGATCTGGTCTCCTCTTAGT	5′-CTGTTGGGTGAGCAGGACC 5′-GCTCCTCTCAGGCAACGGA
+1 Syp −1 Syp +1 Tacstd2 -1 Tacstd2	5′-GACTGGGCTGTTCCGACGAT 5′-CAAACAGGACTCAGGTTGGC 5′-TCCTACCCAGCCTGATCCTTC 5′-GCAATCTCCCCTGCCTGATT	5′-TTCACCACGTCCATGTCTGC 5′-TCCATTCGGGAGGCTAGAGA 5′-AGCGGTGCTAGATCCAAGCC 5′-TCAATGGAATTTGGGTGGCG

### mRNA expression and RT-PCR

RNA was extracted using the EZ-10 spin column total RNA Extraction Kit (Bio Basic). Complementary DNA was synthesized using an Applied Biosystems high-capacity cDNA Reverse Transcription Kit (Thermo Fisher Scientific). Primers were designed to detect levels of the indicated transcripts, and data were normalized to the geometric mean of β-actin and GAPDH. The list of primer sequences is provided in Table 1.

### Statistics

All analyses were conducted in SPSS version 24. Data with more than two groups were analyzed using one-way ANOVA, with least significant difference *post hoc* test used whenever the omnibus test was significant. When only two groups were compared, we used the independent-samples *t* test. In instances where data were compared across days, we used mixed-measures ANOVA, with test day as the within-group variable and condition as the between-group variable. Significance was set at *p* ≤ 0.05.

## Results

### Hippocampal levels of acetylated, but not total, H2A.Z increase 24 h after fear conditioning

**Figure 1.**
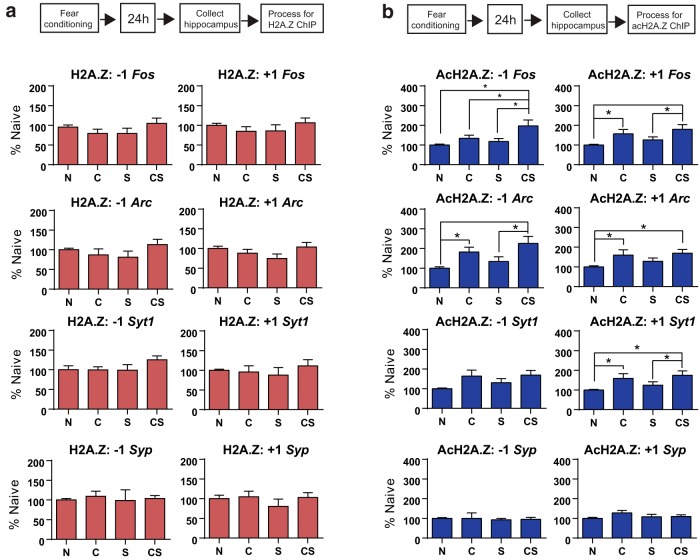
Gene-specific changes in hippocampal H2A.Z and AcH2A.Z binding to memory-related genes 24 h after fear conditioning. ***a***, ***b***, H2A.Z (red; ***a***) and AcH2A.Z (blue; ***b***) binding upstream (−1 nucleosome) and downstream (+1 nucleosome) from the TSS. Data are expressed as the mean ± SEM for each group. N mice, *n* = 10; C mice, *n* = 9; S mice, *n* = 10; CS mice, *n* = 10. *p ≤ 0.05.

To determine whether changes in H2A.Z and acH2A.Z binding to plasticity-related genes occur outside the initial consolidation window (i.e., ∼3 h after learning), we processed hippocampal tissue with ChIP 24 h after learning. Given that H2A.Z is strongly positioned upstream (−1 nucleosome) and downstream (+1 nucleosome) of the transcription start site (TSS; Zovkic et al., 2014; Stefanelli et al., 2018), we focused on these loci. We selected several candidate genes based on their well defined roles in memory, including the immediate-early genes *Arc* and *Fos*. Since recent memory is associated with increased spine density in the hippocampus (Restivo et al., 2009), we also chose to investigate the following two genes that encode synaptic proteins: *Syp*, which encodes synaptophysin; and *Syt1*, which encodes synaptotagmin 1. There were no differences in H2A.Z binding at either the +1 or the −1 nucleosome at any of the genes we investigated (*Fos*, *Arc*, *Syt1*, or *Syp*). In contrast, acH2A.Z binding to several genes was significantly altered by learning. For *Fos*, acH2A.Z binding at the −1 nucleosome was significantly higher (*F*_(3,35)_ = 5.01, *p* = 0.005) in CS mice compared with all other groups (all *p* < 0.05). For the +1 nucleosome, acH2A.Z binding at the *Fos* gene increased with fear conditioning (*F*_(3,35)_ =3.82, *p* = 0.02), whereby CS mice had more acH2A.Z than N (*p* = 0.003) or S (*p* = 0.04) mice (Fig. 1*a*).

Similar findings were observed for acH2A.Z binding at the −1 (*F*_(3,35)_ = 5.09, *p* = 0.005) and +1 (*F*_(3,34)_ = 4.44, *p* = 0.01) nucleosome positions of the *Arc* gene. At the −1 nucleosome, CS mice had higher acH2A.Z than N (*p* = 0.001) and S (*p* = 0.01) mice and C mice had higher acH2A.Z binding than N controls. At the +1 nucleosome of the *Arc* gene, both CS (*p* = 0.005) and C (*p* = 0.004) mice had higher acH2A.Z compared with N mice, and neither group differed from S mice.

**Figure 2.**
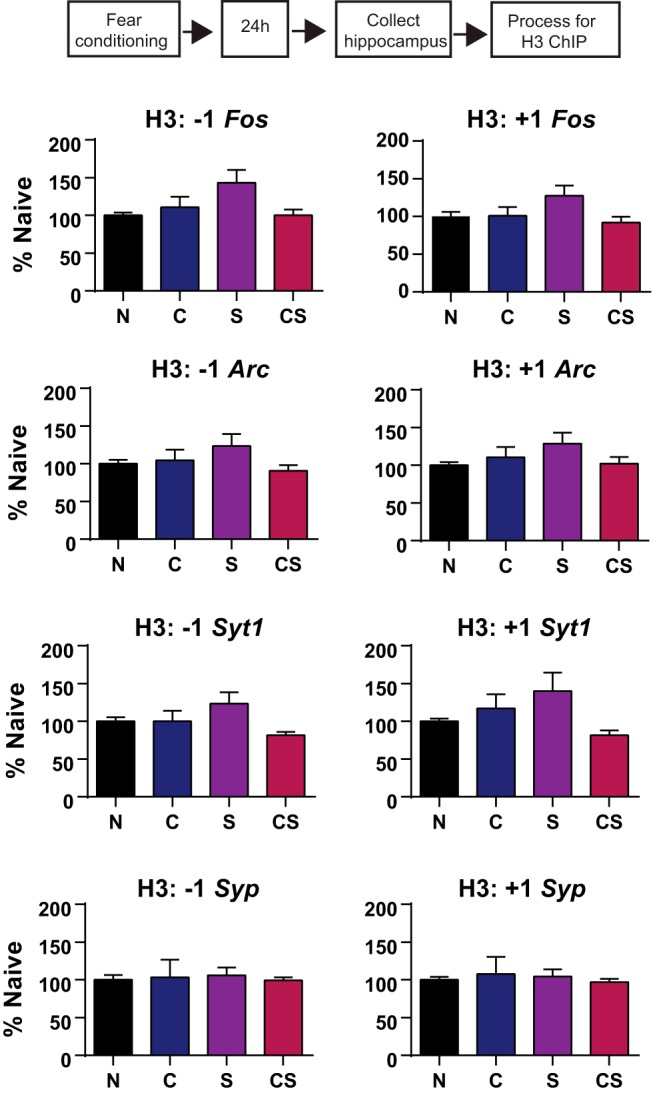
Hippocampal H3 binding at memory-related genes 24 h after fear conditioning. H3 binding is expressed as the mean ± SEM for each group normalized to naive controls. For each gene, data are shown separately for sites upstream (−1 nucleosome) and downstream (+1 nucleosome) of the TSS.

For *Syt1*, there were no differences in acH2A.Z binding to the −1 nucleosome, but there was an increase at the +1 nucleosome (*F*_(3,35)_ = 3.53, *p* = 0.03), with higher levels of acH2A.Z binding to the +1 nucleosome of *Syt1* in CS compared with N mice (*p* = 0.005) and S (*p* = 0.05) controls. In addition, C mice had higher acH2A.Z binding at the +1 nucleosome of *Syt1* compared with N mice (*p* = 0.03). There were no differences in binding for *Syp*. To address any potential changes in nucleosome density, we also quantified H3 binding and found no differences (Fig. 2).

### Stable changes in cortical H2A.Z and acH2A.Z acetylation during memory maintenance

There is extensive evidence that fear memory becomes dependent on the cortex over time, such that fear memory can be recalled even when the hippocampus is lesioned (Frankland and Bontempi, 2005). In support of this hypothesis, recent studies showed that cortical DNA methylation is the only mechanism that exhibits persistent modifications 4 weeks after fear conditioning (Miller et al., 2010; Halder et al., 2016). We previously failed to find stable changes in cortical H2A.Z binding 30 d after training when we focused on immediate early genes (Zovkic et al., 2014), which were recently shown to return to basal levels of DNA methylation during memory maintenance (Halder et al., 2016), prompting us to investigate several novel gene targets. Specifically, we selected several genes that Halder et al. (2016) identified as differentially methylated or upregulated in the cortex 4 weeks after learning, including *Gria4* (encodes AMPA receptor subunit 4), *Tacstd2* (encodes tumor-associated calcium signal transducer 2), and *Syt1* (encodes synaptotagmin 1). These genes also exhibited high levels of H2A.Z binding in our previous ChIP-sequencing study, thus making ideal candidates for our investigation of persistent changes in H2A.Z binding. We also included other genes that exhibit high levels of H2A.Z binding, including *Syp* (encodes synaptophysin) and *Kcna2* (encodes the potassium voltage-gated channel, subfamily A, member 2).

**Figure 3.**
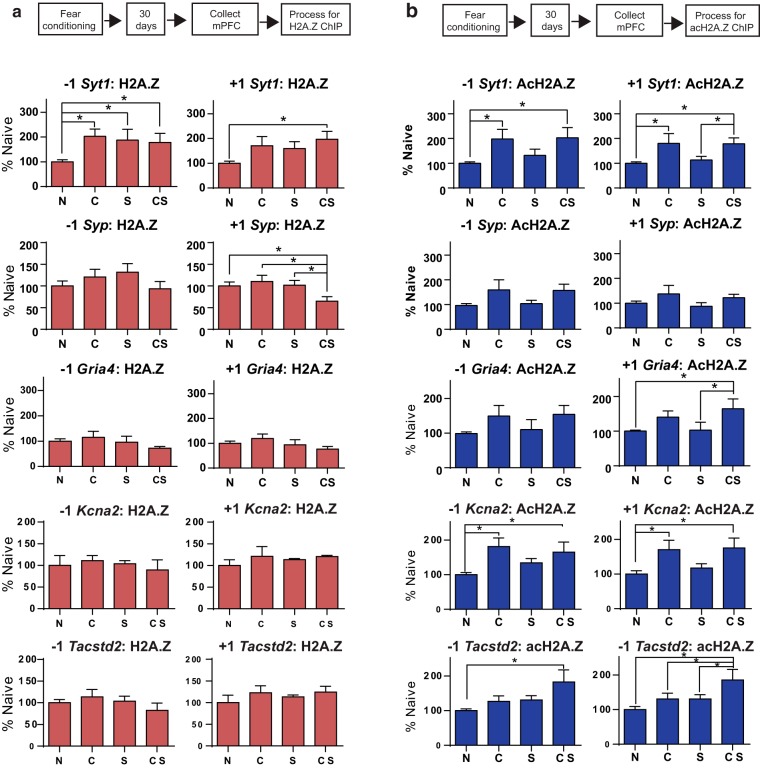
Gene-specific changes in cortical H2A.Z and AcH2A.Z binding to memory-related genes 30 d after fear conditioning. ***a***, ***b***, H2A.Z (***a***) and AcH2A.Z (***b***) binding in the cortex is shown for sites upstream (−1 nucleosome) and downstream (+1 nucleosome) of the TSS. Data are expressed as the mean ± SEM. N mice, *n* = 13–15; C mice, *n* = 6–10; S mice, *N* = 8–9; CS mice, *n* = 10–11. *p ≤ 0.05.

**Figure 4.**
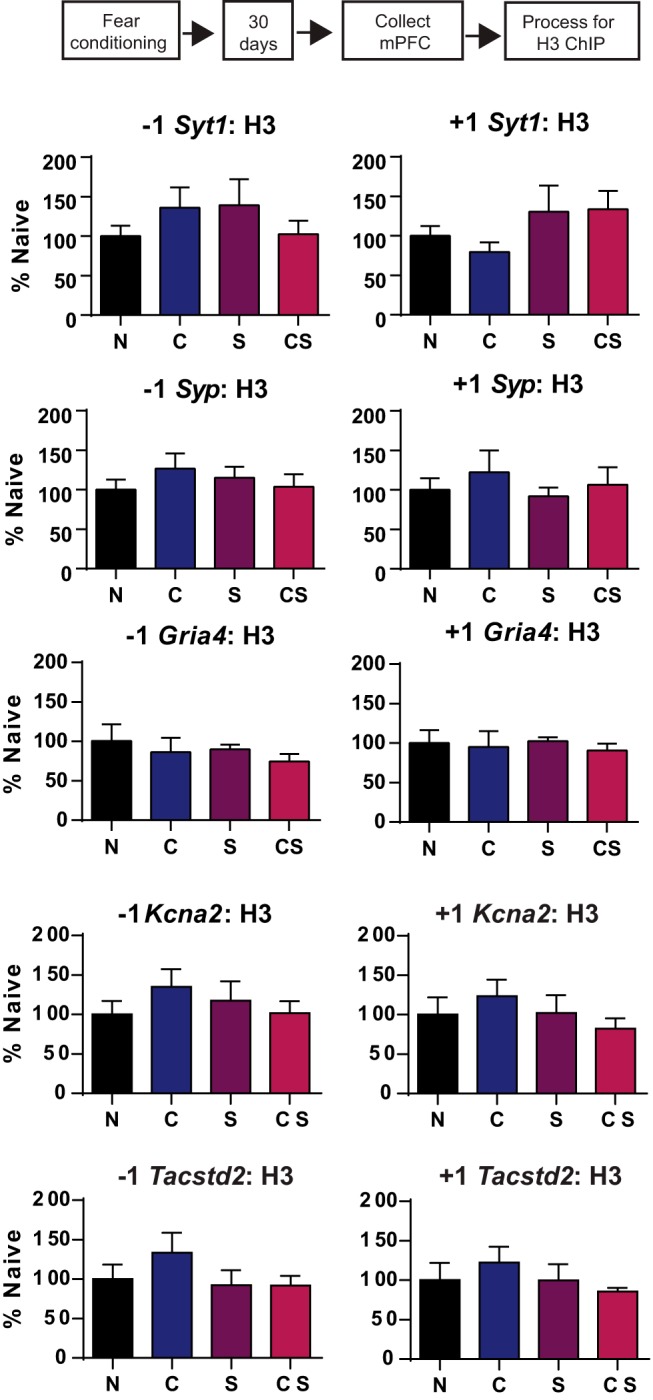
Cortical histone H3 binding at memory-related genes 30 d after fear conditioning. H3 binding is expressed as the mean ± SEM for each group normalized to naive controls. For each gene, data are shown separately for sites upstream (−1 nucleosome) and downstream (+1 nucleosome) of the TSS.

Of the five genes we selected, two showed altered levels of H2A.Z binding. *Syt1* had increased H2A.Z binding at the −1 nucleosome (*F*_(3,36)_ = 2.92, *p* = 0.05), with all groups showing higher levels of H2A.Z binding than N mice (all *p* < 0.05). H2A.Z binding also increased at the +1 nucleosome of *Syt1* (*F*_(3,33)_ = 3.44, *p* = 0.03), with higher H2A.Z occupancy in CS than in N mice (*p* = 0.004). For *Syp*, H2A.Z binding was decreased at the +1 nucleosome (*F*_(3,41)_ = 3.20, *p* = 0.03), with less H2A.Z bound to this position in CS compared with N (*p* = 0.02), S (*p* = 0.03) and C (*p* = 0.01) mice (Fig. 3). To determine whether any changes in H2A.Z binding reflect altered nucleosome occupancy at the gene positions we investigated, we conducted additional analyses of H3 binding and showed no differences across groups (Fig. 4).

In contrast to the relatively restricted changes in H2A.Z binding, changes in acH2A.Z binding to candidate genes were more extensive. For *Syt1*, acH2A.Z binding increased at the −1 (*F*_(3,45)_ = 3.52, *p* = 0.02) and +1 (*F*_(3,46)_ = 3.92, *p* = 0.01) nucleosome. At the −1 nucleosome, CS (*p* = 0.01) and C (*p* = 0.01) mice had more acH2A.Z binding than N mice, and at the +1 nucleosome, CS had more acH2A.Z binding than N (*p* = 0.01) and S (*p* = 0.05) mice. Additionally, C had more acH2A.Z binding than N (*p* = 0.01) and S (*p* = 0.05) mice. AcH2A.Z binding was not altered for *Syp*.

On *Kcna2*, acH2A.Z binding increased at both the −1 (*F*_(3,20)_ = 3.03, *p* = 0.05) and +1 (*F*_(3,20)_ = 3.15, *p* = 0.05) nucleosomes. At the −1 nucleosome, CS (*p* = 0.04) and C (*p* = 0.01) mice had more acH2A.Z than N mice, and, similarly, CS (*p* = 0.02) and C (*p* = 0.03) mice had more acH2A.Z at the +1 nucleosome compared with N mice (Fig. 3).

Finally, ANOVA for *Tacstd2* revealed a trend for increased acH2A.Z binding at the −1 nucleosome (*F*_(3,20)_ = 2.92, *p* = 0.06) and a significant increase at the +1 (*F*_(3,30)_ = 4.01, *p* = 0.02) nucleosome. At the −1 nucleosome, CS had more acH2A.Z binding than N mice (*p* = 0.009) and at the +1 nucleosome, CS had more acH2A.Z binding than N (*p* = 0.003) and S (*p* = 0.007) mice, with a trend for higher binding compared with C mice (*p* = 0.06; Fig. 3). Overall, these data show that the binding of both H2A.Z and acH2A.Z to genes that are implicated in memory maintenance can be stably altered in the cortex, implicating H2A.Z in all phases of memory formation and storage.

### Blocking Tip60 decreases H2A.Z levels and binding *in vitr*o

**Figure 5.**
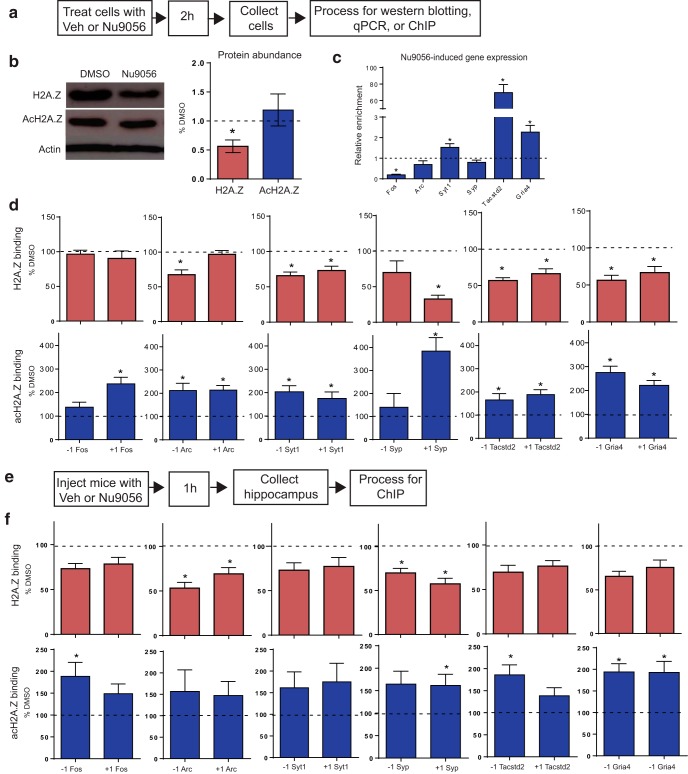
Blocking Tip60 via Nu9056 inhibits H2A.Z expression and binding. ***a***, Outline of the Nu9056 treatment protocol in cultured hippocampal neurons. ***b***, Representative image of immunoblotting for H2A.Z and acH2A.Z (left) and protein quantification (right). Dashed line represents the average of DMSO-treated control neurons (*n* = 3 wells/group). ***c***, Gene expression changes in response to Nu9056 treatment in cultured hippocampal neurons. Dashed line represents the average of DMSO-treated control neurons (*n* = 6 wells/group). ***d***, H2A.Z (top) and AcH2A.Z (bottom) binding at −1 (left) and +1 (right) nucleosomes in neurons treated with Nu9056 (*n* = 6 wells/group). ***e***, Outline of Nu9056 treatment protocol in mice. ***f***, H2A.Z (top) and AcH2A.Z (bottom) binding at −1 (left) and +1 (right) nucleosomes in the hippocampus 1 h after Nu9056 injection (*n* = 7 mice/group). Data are expressed as the mean ± SEM relative to DMSO controls. *p ≤ 0.05. Veh, vehicle.

Data presented thus far show that distinct stages of memory formation and maintenance are each associated with changes in H2A.Z and AcH2A.Z binding to plasticity-related genes. We previously showed that adeno-associated virus-mediated H2A.Z depletion promotes the formation of both recent and remote memory (Zovkic et al., 2014; Stefanelli et al., 2018), but viral manipulations lack the temporal specificity required to assess the effects of H2A.Z within a specific time window, such that mice in our previous studies had reduced H2A.Z levels before learning and throughout subsequent recall testing. To achieve rapid changes in H2A.Z and acH2A.Z levels, we searched for a pharmacological strategy to influence H2A.Z binding. We searched for commercially available drugs with the potential to target H2A.Z and identified the Tip60 inhibitor Nu9056, based on evidence that the Tip60–p400 complex deposits H2A.Z into chromatin (Latrick et al., 2016). As such, we reasoned that Nu9056 would reduce H2A.Z binding by inhibiting Tip60. To test this hypothesis, we treated cultured hippocampal neurons with Nu9056 and measured protein levels of H2A.Z and acH2A.Z using Western blots (Fig. 5*a*). Global levels of H2A.Z (*t*_(4)_ = 2.7, *p* = 0.05), but not acH2A.Z, were reduced 2 h after applying Nu9056 (Fig. 3*b*).

To determine whether Tip60 inhibition affects gene expression, we investigated the effects of Nu9056 on transcription in cultured hippocampal neurons. Nu9056 treatment affected the expression of all genes we investigated, except for *Arc* and *Syp*. Specifically, Nu9056 resulted in decreased levels of *Fos* (*t*_(10)_ = 3.98, *p* = 0.003) and increased levels of *Syt1* (*t*_(10)_ = 3.00, *p* = 0.01), *Tacstd2* (*t*_(10)_ = 1.03, *p* < 0.0001), and *Gria4* (*t*_(10)_ = 3.85, *p* = 0.003), indicating that Tip60 inhibition produces gene-specific effects on transcription (Fig. 5*c*).

We next conducted experiments to confirm that globally decreased H2A.Z levels translate to altered H2A.Z binding on memory-related genes. Cultured hippocampal neurons were again treated with Nu9056 and processed for ChIP to assess H2A.Z and acH2A.Z binding. In agreement with our immunoblotting data, H2A.Z binding was reduced at the −1 nucleosome of *Arc* (*t*_(11)_ = 3.62, *p* = 0.004), *Syt1* (*t*_(11)_ = 5.12, *p* < 0.0001), *Tacstd2* (*t*_(11)_ = 3.89, *p* = 0.003), and *Gria4* (*t*_(11)_ = 6.50, *p* < 0.0001); and the +1 nucleosome of *Syt1* (*t*_(11)_ = 3.53, *p* = 0.005), *Syp* (*t*_(11)_ = 8.18, *p* < 0.0001), *Tacstd2* (*t*_(11)_ = 3.44, *p* = 0.005), and *Gria4* (*t*_(11)_ = 4.44, *p* = 0.001) compared with control neurons treated with DMSO (Fig. 5*d*, top).

In contrast to reduced H2A.Z binding, Nu9056 increased acH2A.Z binding. Specifically, acH2A was increased at the −1 nucleosome of *Arc* (*t*_(11)_ = 3.55, *p* = 0.005), *Syt1* (*t*_(11)_ = 4.19, *p* = 0.002), *Tacstd2* (*t*_(11)_ = 2.59, *p* = 0.03), and *Gria4* (*t*_(11)_ = 6.25, *p* < 0.0001), and at the +1 nucleosome of *Fos* (*t*_(11)_ = 5.28, *p* < 0.0001), *Arc* (*t*_(11)_ = 5.55, *p* < 0.0001), *Syt1* (*t*_(11)_ = 2.81, *p* = 0.02), *Syp* (*t*_(3)_ = 6.36, *p* = 0.01), *Tacstd2* (*t*_(11)_ = 4.18, *p* = 0.002), and *Gria4* (*t*_(11)_ = 5.80, *p* < 0.0001) compared with DMSO-treated neurons. Overall, these data demonstrate that Tip60 inhibition is an effective tool for rapidly reducing H2A.Z binding, while simultaneously increasing acH2A.Z binding (Fig. 5*d*, bottom).

### Tip60 inhibition reduces H2A.Z and increases acH2A.Z binding in the hippocampus

To confirm the efficacy of Tip60 inhibition on H2A.Z and acH2A.Z binding *in vivo*, mice were injected with Nu9056 and the hippocampus was collected 1 h later (Fig. 5*e*). As with cultured neurons, Tip60 inhibition reduced H2A.Z binding at the −1 nucleosome of *Arc* (*t*_(11)_ = 3.44, *p* = 0.006) and *Syp* (*t*_(11)_ = 2.39, *p* = 0.04), and the +1 nucleosome of *Arc* (*t*_(11)_ = 2.18, *p* = 0.05) and *Syp* (*t*_(11)_ = 3.20, *p* = 0.008). Additionally, Nu9056 increased acetylation at the −1 nucleosome of *Fos* (*t*_(12)_ = 2.62, *p* = 0.02), *Tacstd2* (*t*_(12)_ = 2.65, *p* = 0.02), and *Gria4* (*t*_(12)_ = 4.66, *p* = 0.001), and the +1 nucleosome of *Gria4* (*t*_(12)_ = 3.39, *p* = 0.005; Fig. 3*f*).

### Tip60 inhibition 23 h after fear conditioning promotes older (7 d), but not recently acquired (24 h) memory

**Figure 6.**
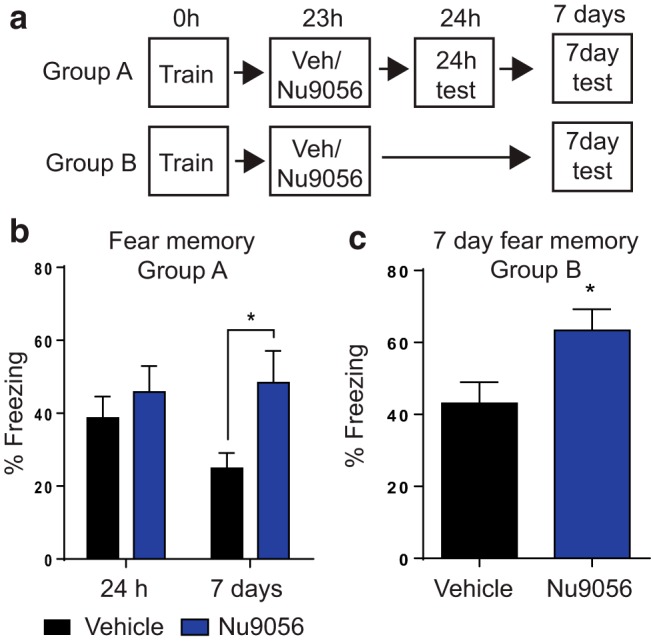
Blocking Tip60 has stage-specific effects on memory. ***a***, Outline of experimental design. Fear memory is indexed as a percentage of the time spent freezing. ***b***, The Tip60 inhibitor Nu9056 does not affect memory when tested 24 h after learning (i.e., 1 h after injection), but promotes memory when tested 7 d after learning (i.e., 6 d after injection) when the same mice are tested at both time points. ***c***, Nu9056 improved remote memory in a separate group of mice that were not tested at 24 h (i.e., recall was tested for the first time 7 d after training/6 d after injection). Data are expressed as the mean ± SEM. Vehicle (Veh), *N* = 12; Nu9056, *N* = 11. *p ≤ 0.05.

Previous studies have shown that manipulations of gene expression between 8 and 24 h after learning selectively impair older (7 d after learning) memories, while sparing newly acquired memories (48 h after learning; Katche et al., 2010, 2012, 2013), indicating that delayed changes in H2A.Z may selectively impact the recall of older memories. To test this hypothesis, we investigated the functional outcomes of Tip60 inhibition 23 h after training on new (tested 1 h later) and older (tested 7 d later) fear memory (Fig. 6*a*). Mice injected with Nu9056 23 h after training did not differ from vehicle-treated controls when tested at 24 h, but the same mice had enhanced memory when tested again 7 d after learning (*F*_(1,21)_ = 6.11, *p* = 0.02), which is when memory becomes increasingly dependent on the cortex (Fig. 6*b*). These data suggest a selective role for delayed Tip60 inhibition in promoting systems consolidation, which is consistent with evidence that delayed gene expression selectively regulates remote memory (Katche et al., 2010, 2012, 2013).

However, we cannot exclude the possibility that differences in memory recall at 7 d are the result of unique effects of Nu9056 on repeated testing. To address this issue, a separate group of mice underwent an identical injection protocol 23 h after training, but were not tested for the first time until 7 d after learning. As with mice that were tested at both time points, Tip60 inhibition at 23 h resulted in improved memory after 7 d (*F*_(1,21)_ = 5.98, *p* = 0.02), suggesting a selective role for hippocampal Tip60 in promoting systems consolidation (Fig. 6*c*).

### HDAC inhibition 24 h after training does not affect remote memory

**Figure 7.**
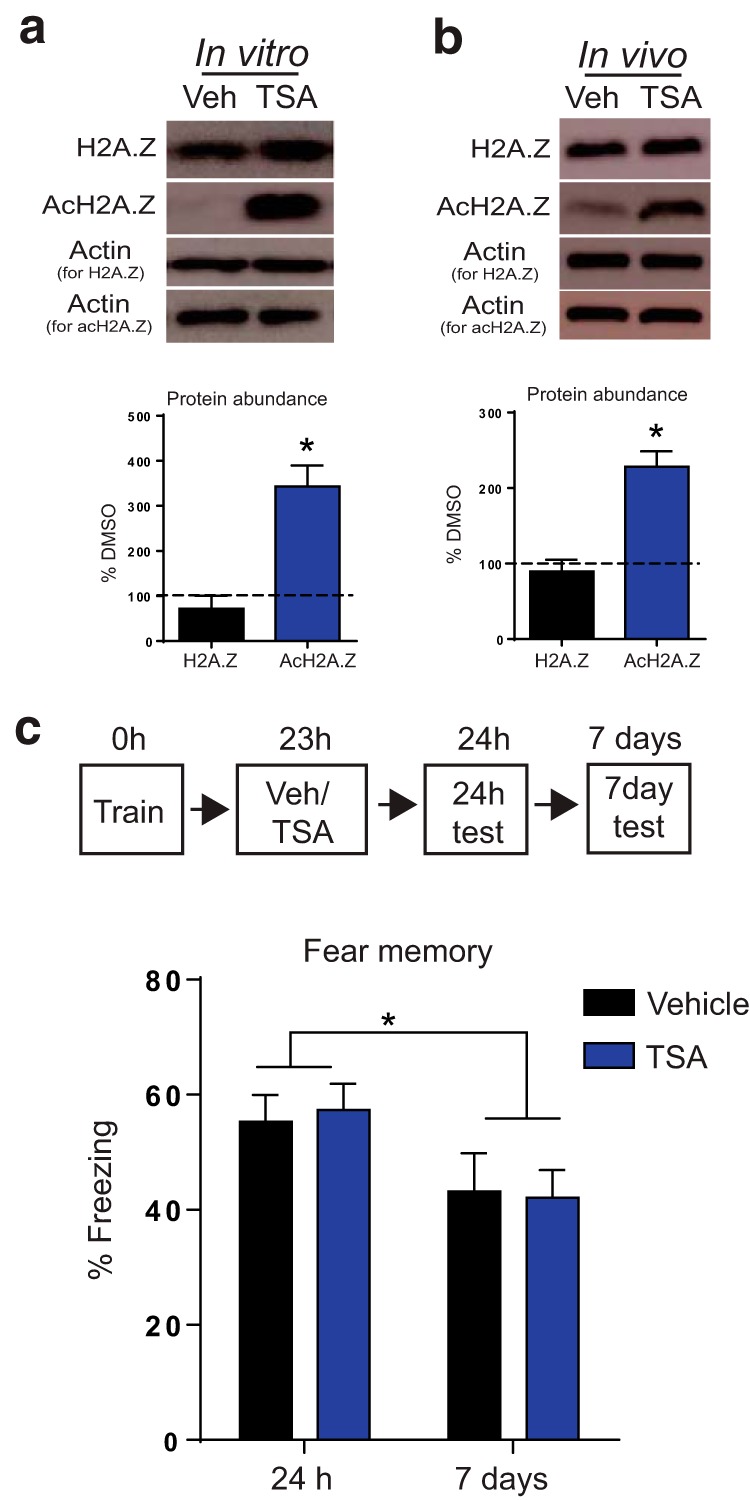
TSA increases H2A.Z acetylation, but does not affect remote memory. ***a***, ***b***, Immunoblot showing levels of H2A.Z and AcH2A.Z after treatment with TSA in cultured hippocampal neurons (*N* = 3 wells/group; ***a***) and adult mouse hippocampus after systemic injection of TSA (*N* = 4 mice/group; ***b***). Separate actin is used for normalizing H2A.Z and acH2A.Z expression because each protein was run on a separate gel to facilitate visualization of similar size proteins. ***c***, Outline of experimental design (top), and memory recall for recent (24 h) and remote (7 d) time points (*n* = 12/group). Data are expressed as the mean ± SEM. *p ≤ 0.05.

Although Tip60 inhibition reduced H2A.Z levels, it also promoted gene-specific H2A.Z acetylation, raising the possibility that Nu9056 influenced memory through changes in acetylation rather than H2A.Z binding. To address this, we searched for alternative methods of increasing acH2A.Z without influencing total H2A.Z levels. Using cultured hippocampal neurons, we showed that TSA, a well characterized HDAC inhibitor, increased H2A.Z acetylation (*t*_(4)_ = 5.22, *p* = 0.006) without altering total H2A.Z (Fig. 7*a*). These results were confirmed *in vivo*, whereby TSA injections resulted in increased levels of acH2A.Z in the hippocampus 1 h later (*t*_(6)_ = 5.53, *p* = 0.002), without altering the levels of total H2A.Z (Fig. 7*b*). Next, we repeated the same behavioral protocol as with Nu9056 and found that, in contrast to Tip60 inhibition, TSA did not affect newly the acquired memory, or an older memory (Fig. 7*c*). This finding is distinct from studies that administer TSA within the initial consolidation window, when it promotes newly acquired memories (Krämer et al., 2003; Korzus et al., 2004; Lattal et al., 2007; Vecsey et al., 2007; Monsey et al., 2011). Indeed, the only significant difference we found was a decrease in freezing across test days (main effect of day: *F*_(1,22)_ = 11.85, *p* = 0.002). Overall, these data suggest that additional enhancement of histone acetylation 24 h after training does not promote either recent or remote memory.

### Tip60 inhibition 30 d after learning has temporally specific effects on remote memory

**Figure 8.**
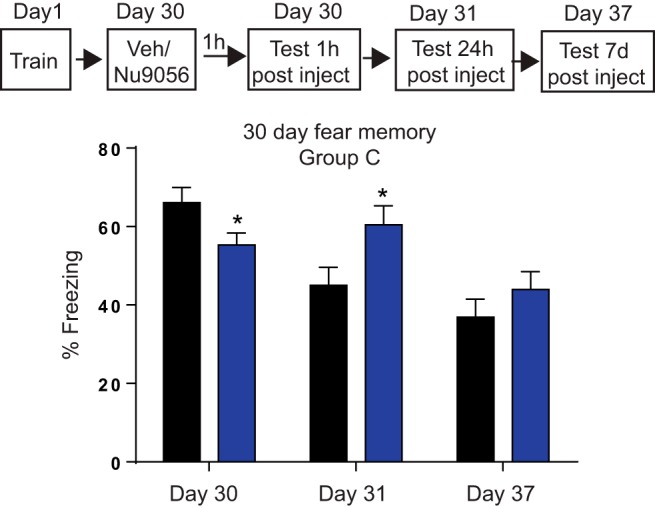
Blocking Tip60 has stage-specific effects on memory. Outline of experimental design is shown on top. Fear memory is indexed as the percentage of time spent freezing. Data are expressed as the mean ± SEM. Vehicle (Veh), *N* = 9; Nu9056, *N* = 10. *p ≤ 0.05.

We previously showed that H2A.Z depletion in the cortex influences memory 7 d, but not 24 h, after learning (Zovkic et al., 2014), indicating that cortical contribution to memory recall becomes evident by 7 d. However, remote fear memories are widely considered to enter the maintenance stage at ∼30 d, when they are no longer dependent on the hippocampus (Walters and Zovkic, 2015). Thus, to determine whether Tip60 inhibition is also involved in regulating the maintenance of remote memory, mice were injected with Nu9056 30 d after learning and tested for recall 1 h after the injection. Mice that received the Tip60 inhibitor exhibited significantly reduced levels of freezing 1 h later (*t*_(17)_ = 2.23, *p* = 0.04), suggesting that Tip60 inhibition during the maintenance phase impairs the recall of remote memories. To determine whether this effect is stable over time, mice were tested again 24 h and 7 d after the injection (no additional injection was given during this time). After 24 h, the data were the opposite of what we observed after 1 h, whereby mice that received the Tip60 inhibitor had higher levels of freezing compared with control mice (*t*_(17)_ = 2.27, *p* = 0.036), suggesting that Tip60 inhibition protected memory from being decayed over time. However, differences in freezing were no longer evident 7 d after the injection, suggesting that the effects of Tip60 inhibition on remote memory are transient (Fig. 8).

## Discussion

We previously identified H2A.Z as a memory suppressor whose levels in the hippocampus increase with age (Zovkic et al., 2014; Stefanelli et al., 2018), suggesting that inhibition of this histone may be useful for the treatment of memory decline. Here, we show that H2A.Z and acH2A.Z binding is modified in the hippocampus well outside the initial consolidation window and in the cortex during memory maintenance, thus implicating H2A.Z as a vital candidate for manipulating memory strength at distinct stages of memory. Strikingly, we identified a pharmacological strategy that rapidly reduces H2A.Z levels via Tip60 inhibition, which achieved unique effects on memory, depending on the time point at which the drug was administered. Specifically, our data provide new evidence for the functional relevance of H2A.Z outside of the initial consolidation window, with distinct outcomes when inhibition occurs 24 h versus 30 d after training. During the 24 h window, Tip60 inhibition improved older memories without affecting newly acquired memories, whereas HDAC inhibition had no effect. This is particularly interesting because H2A.Z acetylation was increased 24 h after learning, yet further increasing acetylation via TSA did not offer an additional advantage for memory recall. These findings contrast the memory-enhancing effects of TSA when it is administered during the initial consolidation window (Vecsey et al., 2007) and suggest that Tip60 inhibition in our study improved memory through effects on total H2A.Z rather than its acetylation. Our findings extend recent observations of epigenetic remodeling 24 h after learning (Gupta et al., 2010; Gupta-Agarwal et al., 2012; Duke et al., 2017) to include H2A.Z and provide functional evidence to implicate delayed epigenetic changes in selectively regulating systems consolidation.

Although Tip60 inhibition had no immediate effect when mice were tested 24 h after training (i.e., 1 h after injection), it did disrupt remote memory 1 h after injection. This likely reflects the distinct nature of the processes that are occurring during each time window. Several studies showed that the recall of recently acquired fear memory (typically measured at 24 h), which is dependent on the hippocampus (Frankland et al., 2004; Frankland and Bontempi, 2005), is not affected by drugs that disrupt transcription, translation, or DNA methylation when these drugs are administered outside of the consolidation window (typically 6 h after training; for review, see Walters and Zovkic, 2015). In contrast, administering the same drugs within the first 2 h after learning does influence the recall of recent memory 24 h after learning, suggesting that hippocampus-dependent recall is selectively influenced by molecular events occurring shortly after learning, but is relatively resistant to changes that occur at later phases after learning. This interpretation is consistent with a series of studies from the Medina laboratory, which demonstrated that disrupting transcription of several memory-related genes between 8 and 24 h after training did not influence recent memory recall, but it did influence memory recall 7 d after learning (Katche et al., 2010, 2012, 2013). Thus, in addition to previously published data, our findings suggest that changes that occur 24 h after learning are likely influencing the strengthening of remote memory rather than the recall of recent memory.

In contrast, the inhibition of DNA methylation during remote memory recall does disrupt remote memory (Miller et al., 2010), suggesting that remote memories may be more sensitive to immediate manipulations. This may be partly due to the fact that 30 d after learning, memories have already entered a maintenance stage, whereas 24 h memories reflect both a process of temporary memory storage in the hippocampus and the ongoing process of systems consolidation that transfers memories to the cortex. Since our studies used systemic injections, we cannot attribute our observations to one brain region or another. However, based on the knowledge of ongoing communication between the hippocampus and the cortex during systems consolidation (Lesburguères et al., 2011; Wiltgen and Tanaka, 2013), it is fair to speculate that changes we observed at the 24 h time point likely reflect modifications to this communication, whereas manipulations of Tip60 after 30 d may more selectively influence the memory maintenance process in the cortex.

Interestingly, Tip60 inhibition appears to inhibit the immediate recall of remote memory, which may reflect the temporary disruption of the role of H2A.Z in memory maintenance. Indeed, we found that some genes had increased, whereas other had decreased levels of cortical H2A.Z binding after 30 d, suggesting that the disruption of this balance with Tip60 inhibition negatively impacts fear memory. However, blocking Tip60 at this time point also resulted in a stronger memory 24 h after injection, which indicates that Tip60 inhibition may disrupt memory updating that is caused by the recall test, such that the reduced freezing in control mice was not evident in mice that received the Tip60 inhibitor.

Of note, many of the changes we observed in H2A.Z and AcH2A.Z binding to DNA were found for mice in the context and shock condition, as well as mice in the control learning conditions. Several genome-wide studies reported extensive overlap both in histone modifications and DNA methylation between fear-conditioned mice and the various control groups (Halder et al., 2016; Duke et al., 2017), but this overlap is offset by unique epigenetic profiles on a subset of genes that are specific to the associative learning condition. Indeed, mice are highly responsive to novelty and shock, which produce well known effects on the expression of immediate early genes, but we also showed at least some selectivity in H2A.Z binding for associative learning. For example, H2A.Z binding was selectively altered in the associative-learning condition for *Syp* and *Tacstd2*, suggesting that this histone is relevant for both nonspecific and associative forms of learning.

Given that the Tip60–p400 complex deposits H2A.Z (Altaf et al., 2010; Gursoy-Yuzugullu et al., 2016; Latrick et al., 2016), the most parsimonious explanation for reduced H2A.Z binding observed with Tip60 inhibition is that inhibition of Tip60 disrupts the Tip60–p400 complex. However, Tip60 also acts as a HAT (histone acetyltransferase) for several histones, including H2A.Z (Kusch et al., 2004; Keogh et al., 2006; Murr et al., 2006; Sun et al., 2009), yet acH2A.Z binding is increased at the same sites at which H2A.Z is reduced. Similar observations were reported in prostate cancer cells during transcriptional induction (Valdés-Mora et al., 2012), suggesting that H2A.Z removal may somehow promote increased acetylation of remaining H2A.Z. Although H2A.Z HATs are not well characterized, there is evidence that the Gcn5-containing complex SAGA [homologous with human P/CAF (P300/CBP-associated factor)] acetylates H2A.Z in yeast (Babiarz et al., 2006; Millar et al., 2006), providing an alternative pathway by which H2A.Z acetylation may be occurring in our study.

The effects of Tip60 on H2A.Z binding in the brain has implications for recent studies showing that Tip60 regulates neural plasticity, presumably through its role as a HAT (Xu et al., 2016; Uchida et al., 2017; Panikker et al., 2018). Our data implicate H2A.Z deposition as another downstream target of Tip60 that may mediate some of its previously reported effects in the brain. These data also reinforce the complexity of epigenetic effects on behavior. For example, TSA is traditionally studied for its effects on the acetylation of canonical histones, but it also affects nonhistone proteins (Singh et al., 2010), and, as our evidence suggests, it dramatically impacts H2A.Z acetylation. Given this complexity, our use of pharmacological inhibitors suggests caution in concluding that Tip60 inhibition impacts remote memory primarily through effects on H2A.Z. However, our conclusion is reinforced by the fact that Tip60 inhibition reduced H2A.Z binding and enhanced remote memory in a manner that is consistent with our prior observations with viral H2A.Z depletion (Zovkic et al., 2014; Stefanelli et al., 2018). Moreover, the inability of TSA to enhance memory despite increased acetylation suggests that the HAT activity of Tip60is not a key contributor to its effects on memory.

Overall, our findings suggest that H2A.Z participates in memory throughout the prolonged process of systems consolidation and memory maintenance. Moreover, pharmacological inhibitors of Tip60 can be used to rapidly reduce H2A.Z levels and improve memory after it has already been formed. These data suggest that ongoing epigenetic events outside of the initial consolidation window influence systems consolidation and that manipulating H2A.Z after a memory already formed can promote its maintenance over time, which has important therapeutic implications for memory decline.

## Synthesis

Reviewing Editor: Cheryl Sisk, Michigan State University Decisions are customarily a result of the Reviewing Editor and the peer reviewers coming together and discussing their recommendations until a consensus is reached. When revisions are invited, a fact-based synthesis statement explaining their decision and outlining what is needed to prepare a revision will be listed below. The following reviewer(s) agreed to reveal their identity: Gleb Shumyatsky, Farah Lubin. Note: If this manuscript was transferred from JNeurosci and a decision was made to accept the manuscript without peer review, a brief statement to this effect will instead be what is listed below.

Please consider including a table that compiles all the gene expression data for each gene tested, which would include time of testing, total gene expression change (or not) and the changes in the gene/histone modifications. Reviewers thought this would enhance the manuscript.
